# Derivatization of Aminoglycoside Antibiotics with Tris(2,6-dimethoxyphenyl)carbenium Ion

**Published:** 2016

**Authors:** A.P. Topolyan, M.A. Belyaeva, E.E. Bykov, P.V. Coodan, E.A. Rogozhin, D.A. Strizhevskaya, O.M. Ivanova, A.V. Ustinov, I.V. Mikhura, I.A. Prokhorenko, V.A. Korshun, A.A. Formanovsky

**Affiliations:** Shemyakin-Ovchinnikov Institute of Bioorganic Chemistry, Moscow, 117997, Russia; Gause Institute of New Antibiotics, Moscow, 119021, Russia; Research Institute of Nutrition, Moscow, 109240, Russia

**Keywords:** aminoglycoside antibiotics, mass-spectrometry, trityl cation, HPLC

## Abstract

Detection of aminoglycoside antibiotics by MS or HPLC is complicated, because a) carbohydrate molecules have low ionization ability in comparison with other organic molecules (particularly in MALDI-MS), and b) the lack of aromatics and/or amide bonds in the molecules makes common HPLC UV-detectors useless. Here, we report on the application of a previously developed method for amine derivatization with tris(2,6- dimethoxyphenyl)carbenium ion to selective modification of aminoglycoside antibiotics. Only amino groups bound to primary carbons get modified. The attached aromatic residue carries a permanent positive charge. This makes it easy to detect aminoglycoside antibiotics by MS-methods and HPLC, both as individual compounds and in mixtures.

## INTRODUCTION

Aminoglycosides are a group of bactericidal antibiotics. Aminoglycoside antibiotics display activity mostly against gram-negative aerobes and are most effective against the majority of severe infections (tuberculosis, endocarditis, and septicemia) [1]. The action of these antibiotics does not depend on the reproduction phase of the microorganisms and relies on aminoglycosides’ irreversible binding to the 30S subunit proteins of bacterial ribosomes, thus inhibiting protein synthesis in bacteria. However, aminoglycosides are poorly active in anaerobic environments, and that makes them ineffective in tissues with reduced circulation and necrotic tissues. The pH of the medium is another factor that influences the antibacterial activity of aminoglycosides: these antibiotics are less effective in acidic and neutral environments than in weakly alkaline conditions. The high ototoxicity and nephrotoxicity [2, 3] associated with aminoglycoside therapy versus other antibiotics is its major shortcoming. Therefore, constant monitoring of aminoglycoside content both in biological fluids and in foods of animal origin is required. Many laboratory assays have been developed over several decades of therapeutic use of aminoglycosides for the detection of these antibiotics using GC-MS, HPLC (including derivatization), ELISA, capillary electrophoresis etc. Two thorough reviews on this area have recently been published [4, 5]. A number of publications [6-18] highlight the importance of and the need for convenient, simple, and rapid procedures for aminoglycoside detection, since the majority of the available techniques are either laborious and time-consuming or use expensive reagents.

**Fig. 1:**
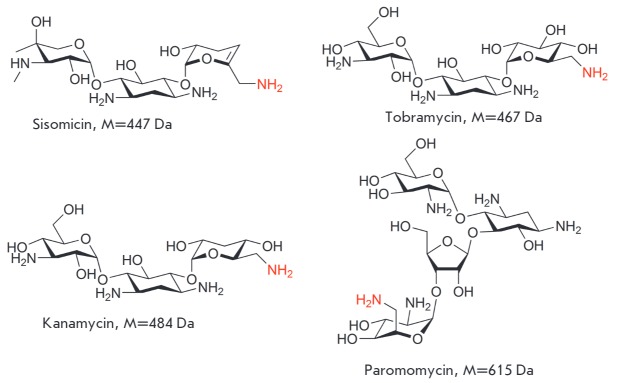
Examples of structures of aminoglycoside antibiotics

The molecules of aminoglycoside antibiotics typically contain several amino groups (*Fig. 1*). These include amino groups directly bound to the heterocyclic or alicyclic ring and amino groups attached to the primary carbon atom (highlighted in red). Another feature of aminoglycosides is the transparency of their solutions in the UV region due to the lack of aromatics and/or amide bonds. This makes common HPLC techniques with UV-detectors not suitable for their analysis. Abundant hydroxyl groups and amino groups capable of forming hydrogen bonds complicate the release of individual molecules, thus leading to a low aminoglycoside ionization ability in mass spectrometry as compared to, for example, peptides of a similar mass. Moreover, aminoglycoside detection in MALDI mass spectrometry can be complicated by matrix interferences.

**Fig. 2:**
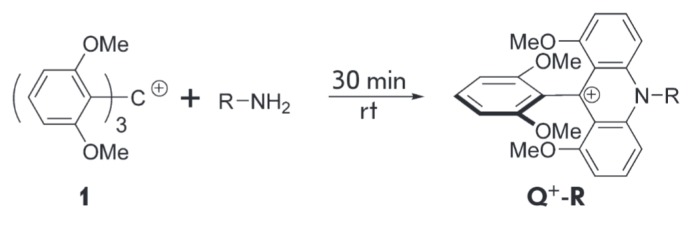
Common scheme of amines derivatization with tris(2,6-dimethoxyphenyl)carbenium

Recently, we have developed a mild derivatization method for low molecular amines using the tris(2,6- dimethoxyphenyl)carbenium ion [19]. The resulting derivatives of the 9,10-disubstituted acridinium cation (**Q^+^-R**) possess a permanent positive charge (*Fig. 2*). Derivatization also results in an increase in mass of the molecule by a constant value (mass increment is +359 Da) that permits successful detection of amines, including those of the smallest mass, with MALDI mass spectrometry [19]. Meanwhile, the modification of hydrophilic aliphatic molecules with hydrophobic aromatic cation Q^+^ may have prospects in terms of reversedphase HPLC with UV detection.

It was of interest to assess the applicability of the derivatization technique for aminoglycoside detection. In this paper, we present a qualitative mass spectrometry and HPLC detection of aminoglycoside antibiotics derivatized with non-cleavable mass tag.

## MATERIALS AND METHODS

**Materials**

Dimethyl sulfoxide and acetonitrile were from Panreac, and other solvents were from Chimmed and EKOS- 1, of chemically pure (cp) grade (hexane, methanol, dichloromethane, ethyl acetate, chloroform, ethanol) and extra-pure grade (toluene, acetone). Dichloromethane was distilled over a calcium hydride, and DMF was distilled over a calcium hydride under vacuum and stored over 3A molecular sieves. Reagents and sorbents included triethylamine, 1,3-dimethoxybenzene, *n*-butylamine (Sigma-Aldrich-Fluka, USA), aminoglycoside antibiotics kanamycin (OAO Biohimik, Saransk, Russia), sisomicin, tobramycin, paromomycin (Minkhimprom, USSR), TLC silica gel aluminum plates (Kieselgel 60 F_254_) or aluminum oxide plates, silica gel, and aluminum oxide (activity I) for column chromatography (Merck, USA).

**Equipment and conditions**

1D and 2D (COSY, HMBC, HSQC) NMR spectra were recorded at 500 MHz (^1^H), 125.7 MHz (^13^C) using the Bruker AC-500 spectrometer and referenced using the residual proton signals of the solvent, DMSO-*d*_6_ (δ_H_ 2.50 ppm and δ_C_ 39.7 ppm) or CD_3_CN (δ_H_ 1.94 ppm for ^1^H and δ_C_ 1.32 ppm); chemical shifts are given with respect to SiMe_4_ (^1^H and ^13^C). TLC-plates were visualized under a UV lamp at 254 and 360 nm. Mass-spectra were recorded using a Ultraflex II TOF/TOF time-of-flight mass analyzer (Bruker Daltonics, Germany) equipped with a nitrogen laser (wavelength of 337 nm) operating at 50 Hz in the positive ion mode with reflectron. Modified aminoglycoside antibiotics were analyzed and separated with preparative reversed-phase HPLC in the acetonitrile gradient using the Agilent Technologies 1200 Series system and Synergi polar-RP reversedphase column (4.5 × 250 mm) under the following conditions: flow rate 0.9 ml/min, 15–50% 80% MeCN + 0.1% TFA for 30 min, 50–70% for 20 min, 70–90% for 10 min, 90% for 5 min, and isocratic elution for 5 min. Absorption was monitored at 285 nm. Conversion of compound (**1**) into compound (**2**) was analyzed using HPLC in the acetonitrile gradient using the Agilent 1100 Series device with multiwave diode array detection. The stationary phase was a Waters Symmetry C8 reversed-phase column. The following conditions were used: flow rate 1 ml/min, acetonitrile gradient – from 50 to 70% for 20 min, from 70 to 98% for 10 min.

For derivatization and dilution of the analytes and matrix compounds, we used acetonitrile (HPLC-grade, JT Baker), methanol (HPLC-grade, Merck), chloroform (HPLC-grade, Merck), and ultrapure water type I obtained using the system Milli-Q (Millipore). The matrixes included 2,5-dihydroxybenzoic, and sinapic and 1-cyano-4-hydroxycinnamic acids (a solution of 20 mg/ ml in acetonitrile with addition of 0.1% trifluoroacetic acid). Sample solution (0.5 μl) in a mixture with the matrix solution (0.5 μl) was loaded onto the target plate spot (MTP 384 massive target gold plate T, Bruker Daltonics, Germany) and air dried.

**Tris(2,6-dimethoxyphenyl)carbenium hexafluorophosphate (1) [20-22]**

A 2.5 M solution of *n*-butyllithium (30 ml, 76 mmol) was added dropwise to a solution of 1,3-dimethoxybenzene (10.0 g, 72.4 mmol) in 100 ml of tetrahydrofuran with stirring under argon and cooling to –20°C. A solution of diethyl carbonate (2.85 g, 24 mmol) in tetrahydrofuran (10 ml) was slowly added 1 h after and stirred at room temperature for 1 day. The solvent was removed under reduced pressure. Diethyl ether (200 ml), dichloromethane (50 ml), and HPF_6_ (30 ml) were added to the residue with stirring. After 3 h, the solvent was removed under decreased pressure, triturated with 300 ml of diethyl ether, and precipitated violet crystals were collected to yield compound **1 **(31.0 g, 76%). 1H NMR spectrum (CD_3_CN, δ_H_, ppm): 3.55 (s, 18H, OCH_3_), 6.61 (6d, 6H, *J *8.54 Hz), 7.63 (3t, 3H, *J *8.54 Hz). Massspectrum (MALDI, m/z, CHCA): 423.15.

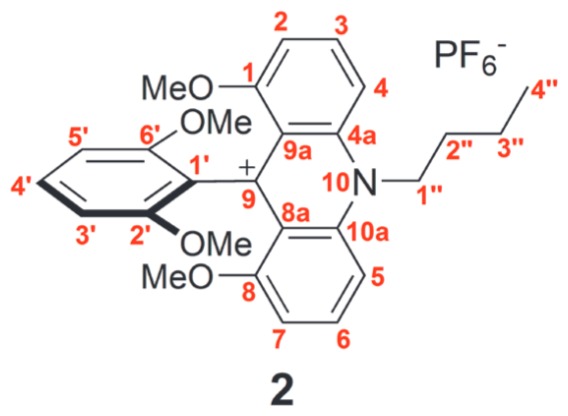



**1,8-Methoxy-9-(2,6- dimethoxyphenyl)-10-(butyl)acridinium hexafluorophosphate (2)**

*n*-Butylamine (350 μl, 3.52 mmol) was added to a solution of tris(2,6-dimethoxyphenyl)carbenium hexafluorophosphate (**1**) (1.0 g, 1.76 mmol) in acetonitrile (15 ml) with stirring at room temperature under argon. The color of the solution changed from purple to red. After 1 h, the solvent was removed under reduced pressure, the solid precipitate was triturated in diethyl ether, and the resulting red precipitate was filtered off and dried in a desiccator in vacuum to give compound **2 **(1.0 g, 98%). 1H NMR spectrum (CD_3_CN, δH, ppm): 1.15 (t, 3H, *J *7.3 Hz, H-4′′), 1.73–1.80 (m, 2H, H-3′′), 2.16–2.22 (m, 2H, H-2′′), 3.57 (s, 6H, OC*H*3,), 3.59 (s, 6H, OCH_3_), 5.06–5.09 (m, 2H, H-1′′), 6.81 (d, 2H, *J *8.5 Hz, H-3′, H-5′), 7.12 (d 2H, *J *8.2 Hz, H-2, H-7), 7.45–7.48 (m, 1H, H-4′), 7.93 (d, 2H, *J *9.2 Hz, H-4, H-5), 8.20–8.24 (m, 2H, H-3, H-6). ^13^C NMR spectrum (CD_3_CN, δ_C_, ppm): 12.96 (4′′), 19.61 (3′′), 29.52 (2′′), 52.37 (1′′), 55.64 (OCH_3_), 56.76 (OCH_3_), 103.74 (3′, 5′), 106.50 (2, 7), 109.26 (4, 5), 119.67 (1′), 119.89 (9), 129.40 (4′), 139.87 (3, 6), 141.61 (1, 8), 155.79 (2′, 6′), 157.18 (8a, 9a), 160.58 (4a, 10a). Massspectrum (MALDI, m/z, CHCA): 432.30.

**A general procedure for derivatization of amino carbohydrates (aminoglucitol, tobramycin, paromomycin, sisomicin) with tris(2,6-dimethoxyphenyl)carbenium hexafluorophosphate**

A relevant amino carbohydrate, 1 eq. in 200 μl of carbonate buffer (pH 9.55), was added to a 0.5 × 10^-2^ M solution of tris(2,6-dimethoxyphenyl)carbenium hexafluorophosphate in acetonitrile (150 μl). The reaction mixture was stirred for 30 min at room temperature. Analysis of the conjugates was carried out directly from the reaction mixture without further purification.

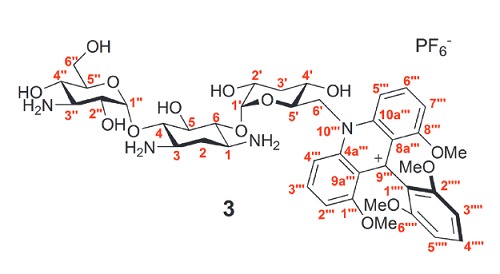



**1,8-Dimetoxy-9-(2,6-dimethoxyphenyl)-10-(6′-deazakanamycin-6′-il)acridinium hexafluorophosphate (3)**

tris(2,6-Dimethoxyphenyl)carbenium hexafluorophosphate (**1**) (2.8 mg, 0.005 mmol) in acetonitrile was added to a solution of kanamycin sulfate (7.8 mg, 0.015 mmol) in 2 ml of buffer solution (pH 9.55). The reaction mixture was kept for 30 min and separated by preparative HPLC. Compound **3 **(10.9 mg, 74%) was obtained. ^1^H NMR spectrum (DMSO-*d*_6_, δ_H_, ppm): 1.69–1.72 (m, 1H, H-2), 2.31–2.33 (m, 1H, H-2), 3.17–3.22 (m, 1H, H-3′′), 3.36 (m, 1H, H-2′), 3.38 (m, 1H, H-1/H-3), 3.40 (m, 1H, H-4′), 3.45 (m, 1H, H-1/H-3), 3.48 (m, 3H, OCH_3_), 3.50 (m, 6H, OCH_3_), 3.51 (m, 1H, H-3′), 3.52 (m, 1H, H-6′′), 3.53 (m, 1H, H-4′′), 3.56 (m, 3H, OCH_3_), 3.57 (m, 1H, H4/6), 3.60 (m, 1H, H-6′′), 3.66 (m, 1H, H4/6), 3.68 (m, 1H, H-2′′), 3.73 (m, 1H, H-5), 3.76 (m, 1H, H-5′′), 4.57– 4.60 (m, 1H, H-5′), 4.74 (s, 1H, OH), 5.03 (d, *J *3.7 Hz, 1H, H-1′′), 5.26 (s, 1H, OH), 5.32 (m, 1H, H-1′), 5.35 (m, 1H, H-6′), 5.55 (m, 1H, H-6′), 6.49 (m, 1H, OH), 6.79–6.83 (m, 2H, H-3′′′′, H-5′′′′), 6.91 (s, 1H, OH), 7.17 (m, 1H, H-2′′′), 7.20 (m, 1H, H-7′′′), 7.42–7.45 (t, 1H, *J *8.5 Hz, H-4′′′′), 8.18 (m, 1H, H-3′′′), 8.19 (m, 1H, H-6′′′), 8.33–8.35 (m, 1H, H-5′′′), 8.45 (m, 1H, H-4′′′). 13C NMR spectrum (DMSO*d*_6_, δ_C_, ppm): 27.48 (2), 46.78 (1/3), 49.28 (1/3), 53.34 (6′), 55.32 (3′′), 55.87 (OCH_3_), 57.06 (OCH_3_), 59.49 (6′′), 65.28 (4′′), 68.39 (2′′), 70.49 (5′), 70.94 (5), 70.98 (2′), 72.33 (4′), 72.58 (3′), 73.08 (5′′), 80.33 (6/4), 83.46 (4/6), 95.66 (1′), 99.28 (1′′), 103.57 (3′′′′/5′′′′), 103.79 (3′′′′/5′′′′), 106.50 (2′′′), 106.75 (7′′′), 110.78 (5′′′), 111.12 (4′′′), 117.60 (1′′′′), 119.21 (2′′′′/6′′′′), 119.30 (2′′′′/6′′′′), 129.19 (4′′′′), 139.08 (3′′′), 139.68 (6′′′), 142.32 (1′′′/8′′′), 143.00 (8′′′/1′′′), 155.65 (9′′′), 156.39 (10a′′′/4a′′′), 158.37 (9a′′′/8a′′′), 159.63 (8a′′′/9a′′′), 159.70 (4a′′′/10a′′′). Mass-spectrum (MALDI, m/z, CHCA): 843.67.

**Derivatization procedure of antibiotics mixture**

We mixed 10 μl of 0.005 M solutions of every antibiotic (kanamycin, sisomin, tobramycin, and paromomycin) in carbonate buffer (pH 9.55), 100 μl of carbonate buffer (pH 9.55), and 50 μl of a 0.005 M solution of salt **1 **in acetonitrile were added. Samples for analysis were taken directly from the reactant mixture.

**Experimental evaluation of the pK_R+_ value of compound 2 [21, 22]**

A solvent system of H_2_O/DMSO/Bu_4_NOH with varying proportions of DMSO and water with a constant concentration of tetrabutylammonium hydroxide (Bu4NOH) was used to assess the pK_R+_ value of compound **2**; its stock solution was added immediately before spectrophotometric measurements. Under strongly basic conditions, the system contains both carbocation R^+^ and its respective non-ionic ROH tritanol with maximum absorption at different wavelengths. The obtained absorbance values in the region of the carbocation absorption maximum (λ = 289 nm) were used to calculate the [R^+^]/[ROH] ratio. The pK_R+_ value was determined from log([R+]/[ROH]) using the *H_ *and *C_ *acidity functions, whose values depend on the molar content of DMSO. Taking into account the measurement error, the measured pK_R+_ value was 18.1 ± 0.5.

**Quantum chemistry calculations**

The structures of the participants of the model transformation mechanism were calculated by the Gaussian- 09 [23] software package with a semi-empirical PM3 method with full optimization of the geometric parameters of the molecules of the reactants and products. The subsequent computation of vibrational frequencies according to the standard procedure of the Gaussian- 09 package showed that the structures meet the criteria of a stationary point (minima and saddle points at the PES). The calculation results were visualized using the ChemCraft program [24].

## RESULTS AND DISCUSSION

**Fig. 3:**
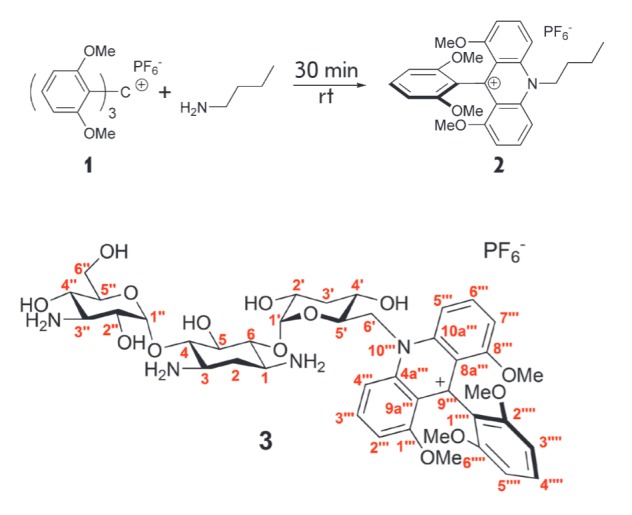
Reaction of **1 **with *n*-butylamine

The reaction of tris(2,6-dimethoxyphenyl)carbenium hexafluorophosphate with *n*-butylamine was studied in order to determine the optimum conditions for amine functionalization (*Fig. 3*).

**Fig. 4:**
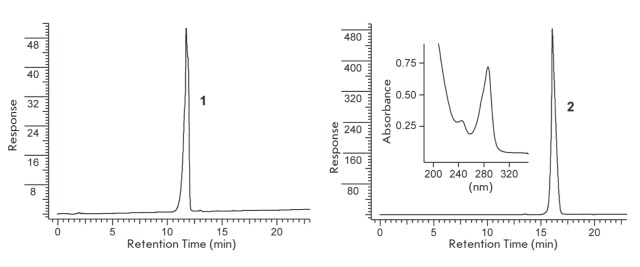
HPLC profile of initial compound **1 **and reactant mixture of **1 **with *n*-butylamine. (see Materials and methods section). Inset – absorption specta of **2**

The full conversion of the initial substrate **1 **into the only product **2 **under excess amine was found to be complete in 10 min in acetonitrile at room temperature. Completeness of the conversion is easily monitored by conventional RP-HPLC as compound **2 **absorbs in the UV range (*Fig. 4*). The reaction does not require any special conditions.

The structure of adduct **2 **was confirmed by 1D and 2D NMR spectroscopy with complete assignment of signals in the ^1^H and ^13^C NMR spectra (see Materials and methods section). The mechanism of compound **2** formation, apparently, involves ipso-attack of the amino group at the ortho-position of a benzene ring followed by elimination of the methoxy group in the form of methanol and repeated nucleophilic substitution on the second ring [21].

In a strongly alkaline medium, the colored cation **2** is able to bind the hydroxide anion to form a colorless tritanol. The pK_R+_ value is a parameter correlating with the stability of the carbocation and corresponding to the pH value at which the concentration of the cationic (colored) form is equal to that of the uncolored form. Based on experimental evaluations, compound **2 **has a value of pK_R+_ ≈ 18, thus indicating the extremely high stability of the cation: the proportion of the cationic form even under mild alkaline conditions is 100%.

**Fig. 5:**
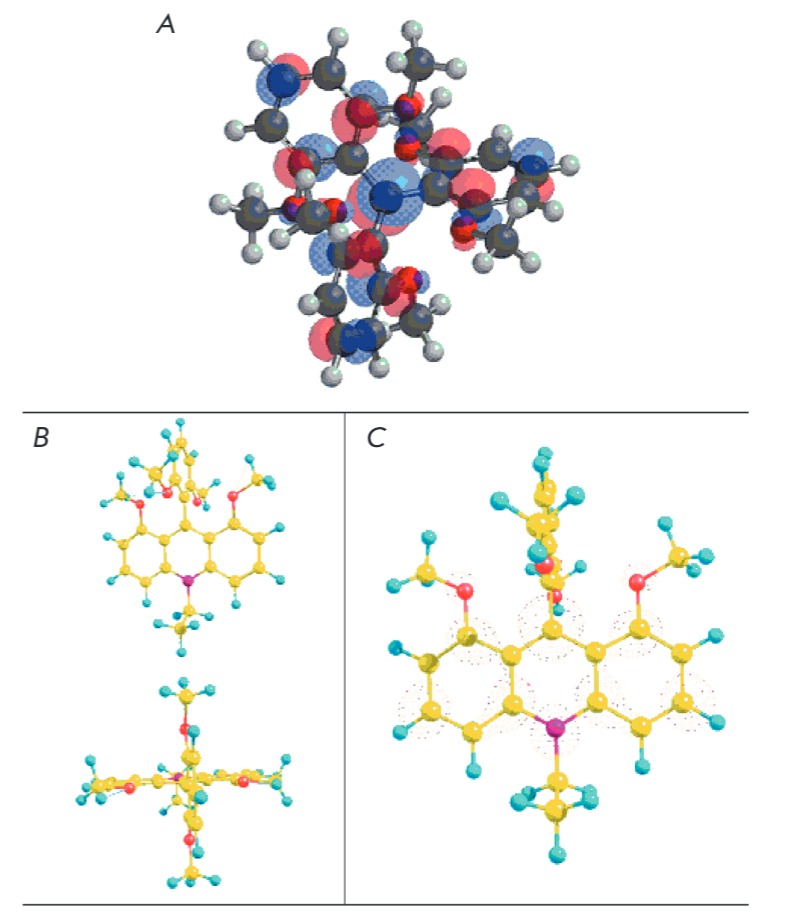
PM3 method calculated *A *– LUMO configuration of cation **1; ***B *– 3D-stucture of cation **Q^+^-Et; ***C *– LUMO configuration of cation **Q^+^-Et. **Carbon atoms – yellow, oxygen atoms – red, nitrogen atoms – pink, hydrogen atoms – turquoise

Quantum-chemical calculation with a semi-empirical PM3 method shows that cation **1 **possesses a propellertype 3D-structure (*Fig. 5A*). The calculated geometric configuration of cation **2 **(with the example of **Q^+^-Et**) is characterized by a marked positioning of the dimethoxyphenyl group in the plane orthogonal to the acridine fragment and a high degree of symmetry (*Fig. 5B*).

**Fig. 6:**
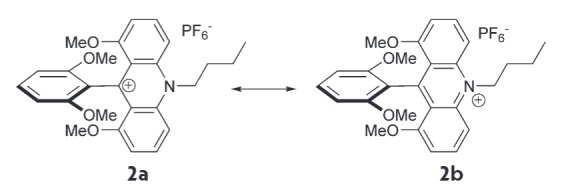
Resonance structures of compound **2**

Formal charges were shown to comprise 0.324 at the C-atom and 0.300 at the N-atom of the central ring of the acridine fragment. The calculated LUMO density at the same atoms (*Fig. 5C*) also coincides with this charge distribution. Thus, the positive charge is predominantly localized on the central carbon atom; thus, the resonance structure **2a **better reflects the structure of substances such as **Q^+^-R** (*Fig. 6*).

**Fig. 7:**
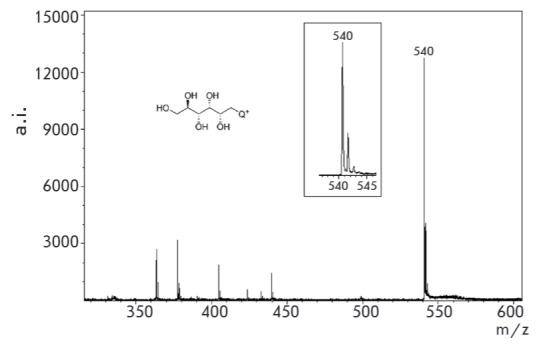
MALDI specta of conjugate **1 **with aminoglucitol (matrix – sinapic acid)

Derivatization of the simplest amino carbohydrate, aminoglucitol, was then studied. Non-derivatized aminoglucitol cannot be detected with MALDI mass spectrometry because of the small molecular weight (181 Da) and poor molecule ionization. TLC monitoring of the original aminoalcohol spot disappearance after subjecting aminoglucitol to an excess amount of the derivatizing agent shows that the reaction is complete within 30 min at room temperature, and the MALDI spectrum demonstrates an explicit signal corresponding to the expected mass of the aminoglucitol conjugate (Fig. 7).

**Fig. 8:**
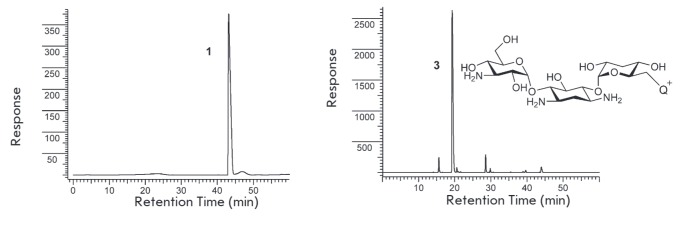
PLC profile of compound **1 **(top) and conjugate **3** (**1 **with kanamycin) (down)

We chose the kanamycin, sisomin, paromomycin, and tobramycin antibiotics (*Fig. 1*) to study the aminoglycoside derivatization. Since the most commonly used form of the kanamycin antibiotic is kanamycin sulfate, the sample was dissolved in carbonate buffer (pH 9.55). As in the case of *n*-butylamine, the reaction proceeds with almost complete conversion (*Fig. 8*).

The structure of the aminoglycosides studied differs by the presence of several amino groups, and any of these can be modified. However, the reaction proceeds smoothly and yields one main product (*Fig. 8*), which was separated by preparative RP-HPLC. Analysis of the 2D-NMR spectra of conjugate **3 **showed that derivatization occurs selectively on the amino group of the primary carbon atom (see Materials and methods section). Probably, this is due to the higher steric accessibility of this amino group versus the amino groups directly attached to the carbon atoms of six-membered rings and shielded with adjacent hydroxyl groups.

**Fig. 9:**
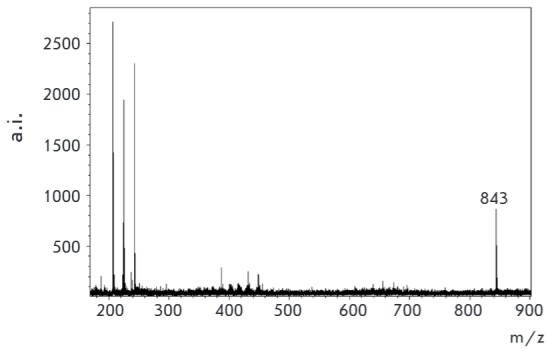
Peak of conjugate **3 **(2×10^-12^ mol of compound per spot) (matrix – sinapic acid) (s/n 47.2)

The derivatization product is easy to detect with mass spectrometry: after loading of 2 × 10^-12^ moles of conjugate** 3 **per spot, a distinct peak of conjugate **3 **with a high signal/noise ratio is observed in the MALDI-MS spectrum (Fig. 9). It should be noted that an increase of the conjugate mass by 359 Da shifts the peak to higher values, which eliminates interferences with matrix signals

**Fig. 10:**
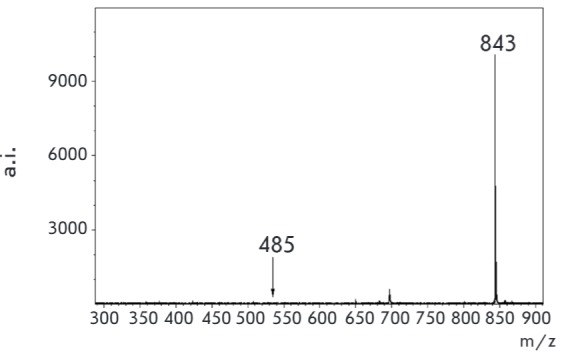
MALDI specta of equimolar mixture of **3 **(m/z 843 (s/n 301.3) and unmodified kanamycin (m/z 485 (s/n 1.8) [M+H^+^]) (matrix – sinapic acid)

With the aim of addressing the effect of derivatization on the detection sensitivity of kanamycin in MALDI- MS, we performed an experiment of simultaneous detection of kanamycin and its derivatization conjugate. *Fig. 10* shows the MALDI-MS spectra of an equimolar mixture of conjugate **3 **and unmodified kanamycin.

**Fig. 11:**
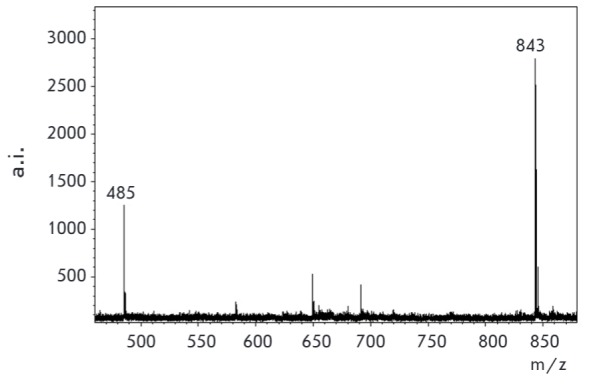
MALDI specta of mixture kanamycin (m/z 485 (s/n 49.1) [M+H^+^]) and conjugate **3 **(m/z 843 (s/n 89.5)) in ratio 200:1 (0.01M : 0.00005 M) (applied 0.9 μL of every sample) (matrix – CHCA)

The trityl/acridine derivative peak intensity is so high that it exceeds the unmodified antibiotic peak intensity by at least two orders of magnitude and visually completely dominates. By increasing the ratio of kanamycin/kanamycin-Q^+^ to 200:1, the signal intensities, becomes similar, but the peak intensity of derivative** 3 **still exceeds that of the unmodified kanamycin (*Fig. 11*). Thus, Q+derivatization reduces the detection limit of kanamycin in MALDI-MS by several orders of magnitude.

**Fig. 12:**
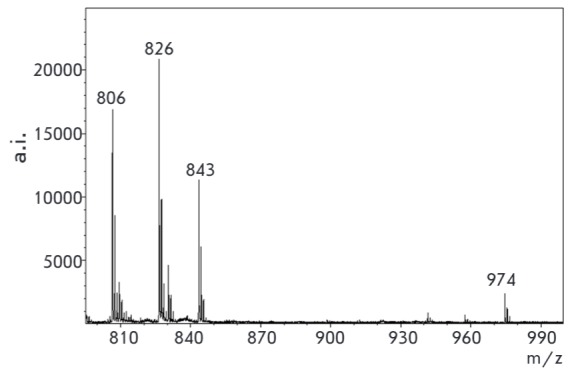
MALDI specta of mixture of modified antibiotics (matrix – sinapic acid) (see Materials and methods section)

When treating kanamycin with an excess of salt **1**, the product of the reaction remains conjugate **3**: the reactivity of other amino groups is considerably inferior to the activity of the -CH_2_NH2 group. This property was used for simultaneous detection of several aminoglycoside antibiotics by mass spectrometry. A mixture of four antibiotics was treated with an excess amount of salt **1, **and the formed adducts were detected in the MALDI spectrum (*Fig. 12*). Peaks of adducts of kanamycin- Q^+^ (**3**) (m/z 843, s/n 142.8), sisomicin-Q+ (m/z 806, s/n 166.4), tobramycin-Q^+^ (m/z 826, s/n 233.2), and paromomycin-Q+ (m/z 974, s/n 56.7) are seen on the spectra.

## CONCLUSIONS

The proposed derivatization method of amine carbohydrates makes it possible to detect them with MALDI mass spectrometry and RP-HPLC with UV detection. The modification is shown to occur at the amino group associated with the primary carbon atom. Derivatization enhances the detection sensitivity of aminoglycosides with mass spectrometry by several orders of magnitude. The speed, simplicity, and the availability of reagents are the advantages of the derivatization method.

## Abbreviations

HPLC: High performance liquid chromatography
GC-MS: gas chromatography–mass spectrometry
ELISA: enzyme-linked immunosorbent assay
MALDI-MS: matrix-assisted laser desorption/ionization mass spectrometry
LUMO: lowest unoccupied molecular orbital
CHCA: 1-cyano-4-hydroxycinnamic acid
TLC: thin-layer chromatography
NMR: nuclear magnetic resonance
DMF: dimethylformamide
DMSO: dimethyl sulfoxide
